# Effect of Carbonation on Chloride Maximum Phenomena of Concrete Subjected to Cyclic Wetting–Drying Conditions: A Numerical and Experimental Study

**DOI:** 10.3390/ma15082874

**Published:** 2022-04-14

**Authors:** Lina Xu, Yan Zhang, Shuyuan Zhang, Shuyuan Fan, Honglei Chang

**Affiliations:** 1 Shandong University of Engineering and Vocational Technology, Jinan 250200, China; cementitious_3d@163.com; 2 Shandong Jinan Rail Design Consulting Co., Ltd., Jinan 250001, China; four_1102@163.com (Y.Z.); nrgzhangyan@126.com (S.Z.); 3 School of Qilu Transportation, Shandong University, Jinan 250002, China; 202135401@mail.sdu.edu.cn

**Keywords:** concrete, chloride distribution, cyclic wetting–drying, carbonation, modeling

## Abstract

The combined action of chloride and carbonation generally accelerates chloride penetration in concrete. Plenty of studies have revealed a chloride maximum phenomenon in the chloride profiles of concrete under wetting and drying cycles, which affects the accuracy of the service life prediction of concrete structures. Carbonation is probably one of crucial factors inducing chloride maximum phenomena. To investigate the influence of carbonation on chloride distribution of concrete subjected to cyclic wetting–drying conditions, this study established a numerical model coupling carbonation effect, simulated chloride distribution at different carbonation degrees, and verified the simulation results with experimental results. The results show that a chloride peak appears in all predicted chloride profiles when carbonation effect is taken into account, and the higher the carbonation degree is, the more significant the chloride peak is. This demonstrates that carbonation can enhance the forming of chloride maximum phenomenon under cyclic wetting and drying. Moreover, the calculated results are highly consistent with the experimental results under different carbonation conditions, especially in terms of the peak chloride concentration and the corresponding depth. Furthermore, the significance degree of the chloride maximum phenomenon is closely related to some key parameters, such as *CO_2_* concentration, environmental humidity, and temperature.

## 1. Introduction

Steel corrosion induced by chloride ingress is one major cause for the deterioration of concrete structures and the curtailment of service life. In recent decades, massive work has been done in the field of chloride transport, including experimental investigations and the establishing of numerical models. In saturated concrete, the dominating mechanism for chloride transport is diffusion initiated by chloride concentration gradient [1,2,3,4,5]. However, in non-saturation state, which is more common, the mechanisms dominating chloride transport in concrete are complicated. Besides diffusion, capillary absorption also drives water, which carries chloride ions to transmit into concrete [5,6,7,8,9]. In addition, chloride attack is generally severer under such a circumstance.

The wetting and drying environment is a typical condition that results in the unsaturation of concrete. In addition, it is widely acknowledged that chloride penetration is the most severe under such a condition due to the combined action of chloride and carbonation. Chloride distribution in concrete is very complex under cyclic wetting–drying conditions. Instead of chloride content decreasing monotonously with increasing distance from the exposed surface, a maximum phenomenon [10,11,12] has been reported by many studies [13,14,15,16,17,18], that is, chloride content first increases, reaches a climax, and then decreases. Affecting the accuracy of the service life prediction of concrete, the maximum phenomenon has gained increasing attention [19,20,21,22,23,24,25]. Capillary suction may be one major cause for the forming of chloride climax during cyclic wetting–drying, as has been supported by many studies [8,26,27,28,29]. Additionally, carbonation is also a crucial factor, though it is easily overlooked. Actually, *CO_2_* in air can directly enter into concrete during drying and initiate carbonation reaction that affects chloride transport, especially in wet–dry conditions of high *CO_2_* concentration, like the entrance of subsea tunnel and the urban deicing environment.

The carbonation process is very complicated, and its influence on chloride distribution is difficult to state clearly. Liu [30] studied the interaction between carbonation and chloride attack in ordinary Portland cement concrete. The results showed that carbonation decreased surface chloride content, changed chloride profile significantly, and greatly expanded the convection zone range. Jonathon [31] explored the impact of carbonation on chloride transport under cyclic wetting and drying, and found that carbonation influenced chloride distribution through significantly changing the chloride binding capacity of paste. Wang and Basheer [32] reported a detailed investigation on the effect of carbonation on chloride penetration in different types of concrete, and found that carbonation could accelerate chloride penetration, but the acceleration rate depended on the carbonation-chloride combined regimes and the binder type. Moreover, the influence of carbonation on the chloride distribution of concrete subjected to wetting and drying cycles were systemically studied by Chang [11], and the results demonstrated that chloride maximum phenomenon occurred both in normal atmosphere environment and high *CO_2_* concentration environment, and that of the latter was more significant. Additionally, many studies have investigated the influence of carbonation through modeling. Yoon [33] and Puatatsananon [34] simulated chloride transport by coupling chloride diffusion and carbonation, focusing on the influence of pore structure variation caused by carbonation on chloride transport. The calculation result showed that carbonation could significantly decrease chloride diffusion rate in concrete. Meijers [35] set up a chloride transport model under cyclic wet–dry environment with the carbonation effect being considered. The predicted results suggested that the chloride maximum phenomenon would appear in chloride profiles as long as the effect of carbonation on bound chloride was taken into account. Besides considering the effect of carbonation on bound chloride, Zhu [36] also coupled the influence of carbonation on porosity, tortuosity, and connectivity, and the acceleration of chloride diffusion, and the maximum phenomenon was also observed in calculated chloride profiles. Shen and Liu [37] investigated the combined ingress of chloride and carbonation in marine-exposed concrete under unsaturated condition through numerical modelling, and also found that carbonation had an important effect on chloride transport. There are plenty other studies [38,39,40,41,42,43] that have probed the influence of carbonation on chloride transport through experiments or modeling. The above studies have made important contributions and laid the foundation for subsequent research. Moreover, it can also be found out that carbonation is an important and non-negligible factor in analyzing the chloride distribution of concrete under cyclic wetting–drying conditions. However, as carbonation is a very complex process, there are still some disagreements between the simulation results and experimental results of different studies, and further studies are still needed.

As a matter of fact, carbonation influences chloride transport mainly in terms of two aspects: chemical effect and physical effect. Chemical effect refers to carbonation decomposing the bound chloride ions adsorbed on hydration products, especially in Friedel’s salt and C-S-H gel [44,45,46,47]. Those decomposed bound chloride ions dissolve in pore solution, which increases the free chloride content and influences chloride transport. Physical effect refers to carbonation products being deposited in the pore structure, leading to low porosity and redistribution of the aperture, which affects chloride distribution [48,49,50,51]. Moreover, the pore structure change induced by carbonation can affect moisture transport in concrete, which in reverse influences carbonation itself, because *CO_2_* diffusion is dependent on the water content of concrete.

As a follow-up to a previous investigation (explored the influence of carbonation on chloride distribution through experiments [11]), this study established a numerical model coupling carbonation effect, simulated chloride distribution at different carbonation degrees, and verified the simulation results with experimental results. To guarantee the accuracy of predicted chloride distribution, it is essential to couple both the chemical and physical effect of carbonation when simulating chloride transport under cyclic wetting and drying conditions. In Section 3, a model coupling both the two effects will be presented with the mathematical formulas and deduction process being elaborated. In Section 4, the accuracy of the simulation will be verified through comparing calculation results with experimental results, to explore the influence of carbonation on chloride distribution and maximum phenomenon.

## 2. Experiments

### 2.1. Raw Materials

P.II 52.5 Portland cement was used to mold mortar specimens. The chemical composition of the cement is presented in Table 1. The mixing water was deionized water, and the sand is river sand with fineness of 2.2. Three different water-to-binder ratios (*w*/*b*) were employed, being 0.3, 0.4, and 0.5, respectively. The cement-to-sand ratio was 1:3. Please note that deionized water was used to avoid incorporation of chloride ions in mortar.

### 2.2. Sample Preparation

The size of the mortar specimens was 70 mm × 70 mm × 70 mm. After casting, the molds were sealed with cling film on the surface to reduce moisture evaporation. Then, mortar specimens were placed in a room of 20 ± 1 °C and over 90% relative humidity for 24 h. Afterwards, they were demolded and cured for 28 days at 20 ± 1 °C. Next, a 20 mm thickness of each mortar specimen was cut off from the mold free surface to guarantee the homogeneity of specimens after curing. Before being subjected to the prescribed exposure conditions, all the faces of the mortar specimens were sealed with epoxy resin except the cutting surface.

### 2.3. Exposure Conditions

Specimens were then exposed to three different wetting and drying conditions: *N_2_* condition, normal air condition, and accelerated carbonation condition. In addition, the detailed information is listed in Table 2. The temperature, humidity, NaCl solution concentration, and wetting and drying time of the three conditions were identical, with temperature being 20 ± 1 °C, humidity 70 ± 1%, NaC1 solution 3.5%, wetting time 6 d, and drying time 1 d. This wetting and drying regime has been widely used in previous studies [52,53,54]. The major difference among them is the varying *CO_2_* concentration. Under the *N_2_* condition, specimens were placed in a sealed container full of *N_2_* with nil *CO_2_*. Under the normal air condition, specimens were exposed to indoor environment of constant humidity and temperature, with *CO_2_* concentration being about 0.04% by volume. In addition, under the accelerated carbonation condition, specimens were placed in an accelerating carbonation chamber with *CO_2_* concentration being 20.0% by volume. Additionally, specimens of all three different *w*/*b* were subjected to the normal air condition, while only specimens of *w*/*b* = 0.5 were exposed to the *N_2_* condition and accelerated carbonation condition.

### 2.4. Free Chloride Content

All specimens were exposed for 12 weeks. After reaching exposure time, a high-precision numerical controlled milling machine introduced in Ref. [55] was used to grind sample powder layer by layer with thickness of each layer being 0.5 mm. Then, the silver nitration titration method was employed to test the free chloride content in compliance with the *Testing Code of Concrete for Port and Waterway Engineering* [56]. In addition, the detailed operation can be found in reference [56].

Please note that the experimental results in this study were obtained from mortar specimens to verify the reliability of the numerical model proposed. The reason mortar specimens rather than concrete were used is that the coarse aggregates in concrete influence chloride distribution in a negative way. If concrete specimens were used, the inhomogeneity caused by the large size coarse aggregates in them may render the failure of presenting maximum phenomenon that had already formed or great deviance between predicted and detected depth of chloride peak, which directly influence the verifying results of the model proposed. However, using mortar specimens can avoid the problems mentioned above. Furthermore, the verification of the model proposed becomes feasible by converting the unit of detected and predicted chloride content into the percentage of cement (% cement).

## 3. Numerical Model

### 3.1. CO_2_ Transport

The governing diffusion equation of *CO_2_* is as follows [36]:

(1) $$\frac{\partial \left(\phi -\theta_{w}\right)C_{CO_{2}}}{\partial t}=-\nabla \cdot J_{CO_{2}}-I_{ch}$$

where $C_{CO_{2}}$ is the molar concentration of gas *CO_2_* in pore (mol/m^3^ pore air); ϕ is the current porosity of concrete (m^3^ pore volume/m^3^ concrete); $\theta_{w}$ is the volume fraction of pore solution or moisture content (m^3^ pore solution/m^3^ concrete); $I_{ch}$ is the consumption rate of *CO_2_* reacting with *Ca(OH)_2_*. In this model, the consumption of *CO_2_* is assumed to be the *CO_2_* consumption reacting with *Ca(OH)_2_*.

$J_{CO_{2}}$ is the flux of *CO_2_* in concrete,

(2) $$J_{CO_{2}}=-D_{CO_{2}}^{car}\nabla C_{CO_{2}}$$

where $D_{CO_{2}}^{car}$ is the diffusion coefficient of *CO_2_*, m^2^/s.

It is generally believed that gas cannot penetrate the aggregates in concrete, and thus the transmission of *CO_2_* is mainly through the pores in cement paste. Besides, the higher the humidity in pores is, the harder it is for *CO_2_* diffusion to take place. Different models for the determination of $D_{CO_{2}}^{car}$ can be found in the literature [57,58]; the following expression proposed by Papadakis [57] was employed.

(3) $$D_{CO_{2}}^{car}=1.64\times 10^{-6}\phi_{p}^{1.8}{\left(1-h\right)}^{2.2}$$

where $\phi_{p}$ is the porosity of hardened paste; *h* is the relative humidity in pores.

$\phi_{p}$ is also closely related to the mix proportion parameters of concrete, and thus the relation between $\phi_{p}$ and ϕ can be presented by Equation (4).

(4) $$\phi_{p}=\phi \left[1+\frac{(a/c)\left(\rho_{c}/\rho_{a}\right)}{1+(w/b)\left(\rho_{c}/\rho_{w}\right)}\right]$$

where a/c is the aggregate-to-cement ratio; w/b is the water-to-binder ratio; $\rho_{a}$ is the density of aggregate, 2580 kg/m^3^; $\rho_{c}$ is the density of concrete, 2300 kg/m^3^; $\rho_{w}$ is the density of water, 1000 kg/m^3^.

Since the pores in concrete will be partially filled by carbonation products during carbonation process [59], ϕ will change with carbonation degree, and their relation can be portrayed by Equation (5).

(5) $$\phi =\phi_{0}-\Delta \phi_{c}\alpha_{c}$$

where $\phi_{0}$ is the porosity of non-carbonated concrete; $\Delta \phi_{c}$ is the ultimate reduction of porosity after complete carbonation; $\alpha_{c}$ is the carbonation degree.

The volume change of porosity during carbonation is mainly related to the types of Ca contained compounds, and thus $\Delta \phi_{c}$ can be obtained through Equation (6).

(6) $$\Delta \phi_{c}=\left({\left[C_{CaO}\right]}_{0}-3{\left[C_{CSH}\right]}_{0}\right)\Delta V_{ch}$$

where ${\left[C_{CaO}\right]}_{0}$ is the initial molar concentration of total CaO in concrete, 2970 mol/m^3^ concrete; ${\left[C_{CSH}\right]}_{0}$ is the initial molar concentration of C-S-H gel in concrete, mol/m^3^ concrete; $\Delta V_{ch}$ is the molar volume change of *Ca(OH)_2_* after reacting with *CO_2_*, 3.85 × 10^−6^ m^3^/mol. The relation of ${\left[C_{CaO}\right]}_{0}$ to ${\left[C_{CSH}\right]}_{0}$ can be presented by Equation (7).

(7) $${\left[C_{CSH}\right]}_{0}=\frac{P_{CSH}{\left[C_{CaO}\right]}_{0}}{3}$$

where $P_{CSH}$ is the proportion of *CaO* in the form of C-S-H gel in total. The value of $P_{CSH}$ is closely related to mix proportion, and 38.8% was adopted in this model.

Generally, the evolution of carbonation degree can be reflected by the consumption of *Ca(OH)_2_* [60] in pore solution.

(8) $$\alpha_{c}=1-\frac{C_{ch,d}}{{\left[C_{ch,d}\right]}_{0}}$$

where $C_{ch,d}$ is the molar concentration of *Ca(OH)_2_* in pore solution, mol/m^3^ pore solution; ${\left[C_{ch,d}\right]}_{0}$ is the initial molar concentration of *Ca(OH)_2_* in pore solution, mol/m^3^ pore solution.

During carbonation process, besides the balance between *CO_2_* diffusion and consumption, *Ca(OH)_2_* in pore solution also remains balanced as presented by the equations below.

(9) $$\frac{\partial \theta_{w}C_{ch,d}}{\partial t}=-\nabla \cdot J_{ch,d}$$

(10) $$J_{ch,d}=-D_{ch,d}\nabla \cdot C_{ch,d}$$

where $J_{ch,d}$ is the flux of dissolved *Ca(OH)_2_*; $D_{ch,d}$ is the diffusion coefficient of dissolved *Ca(OH)_2_*, 1.0 × 10^−13^ m^2^/s.

Moreover, the expression of $I_{ch}$ was selected according to the research of Papadakis [57].

(11) $$I_{ch}=\phi f_{w}r_{c}C_{CO_{2}}H\left(C_{ch,d}\right)$$

where $f_{w}$ is the volume fraction of water film on the wall of pores, being 0.05; $r_{c}$ is the dependence of reaction on the temperature; *H* is the Heaviside step function, and its value is 1 in this study.

$r_{c}$ can be calculated through Arrhenius type equation as follows [57].

(12) $$r_{c}=A_{c}exp\left(\frac{E_{c}}{RT}\right)$$

where $A_{c}$ is the scaling factor of the reaction, 359/s; $E_{c}$ is the activation energy of the reaction, 442 J/mol; R is the gas constant, 8.314 J/(mol⋅K); *T* is the absolute temperature, K.

### 3.2. Moisture Transport

Supposing that concrete under cyclic wet–dry conditions is only subjected to humidity change without water flowing driven by macroscopic pressure, then moisture transport in concrete is all about vapor transmission. Hence, for non-saturation specimens with evenly distributed temperature, the flux of moisture can be presented by Equation (13) [61].

(13) $$j_{w,mass}=-\delta_{v}\left(h\right)p_{v,sat}\nabla h$$

where $j_{w,mass}$ is the flux of moisture, kg/(m^2^·s); *δ_v_(h)* is the water vapor penetration coefficient, s; $p_{v,sat}$ is the water vapor saturation pressure, Pa.

*δ_v_(h)* is controlled by temperature and can be obtained by the equation below [62].

(14) $$\delta_{v}\left(h\right)=\alpha_{0}f_{1}\left(h\right)$$

where $\alpha_{0}$ is the reference penetration coefficient at temperature 25 °C, and its value ranges from 10^−10^ to 10^−14^ s; *f_1_(h)* is the influence function of moisture transport that reflects water transmission within the absorbed water layers [62,63], and it can be represented by Equation (15).

(15) $$f_{1}\left(h\right)=\alpha +\frac{1-\alpha }{1+{\left(\frac{1-h}{1-h_{c}}\right)}^{4}}$$

where *α* ≈ 0.05; *h_c_* ≈ 0.75 at temperature 25 °C.

The pore change caused by carbonation will influence the transport of water vapor. Therefore, when carbonation is taken into consideration, the *δ_v_(h)* equation is transformed into the equation below,

(16) $$\delta_{c}^{car}\left(h\right)=\delta_{v}\left(h\right)f_{p}\left(\Omega ,\delta \right)$$

where $f_{p}\left(\Omega ,\delta \right)$ is the function reflecting the influence of pore structure, and it can be expressed as

(17) $$f_{p}\left(\Omega ,\delta \right)=\frac{\delta }{\delta_{0}}\frac{\Omega_{0}}{\Omega }$$

where δ and Ω are the current constrictivity and tortuosity of pore structure, respectively, which are closely related to pore size and porosity separately; $\delta_{0}$ and $\Omega_{0}$ are the initial constrictivity and tortuosity before carbonation respectively. According to literature [64,65], the empirical formulas of δ and Ω can be expressed as:

(18) $$\Omega =-b_{1}\;tan\;h\left[b_{2}\left(\emptyset -b_{3}\right)\right]+b_{4}$$

(19) $$\delta =-c_{1}\;tan\;h\left[c_{2}\left(logr_{p}+c_{3}\right)\right]+c_{4}$$

where $r_{p}$ is the peak radius at different carbonation degree, m. Moreover, $b_{1}=1.5$, $b_{2}=8.0$, $b_{3}=0.25$, $b_{4}=0.25$, $c_{1}=0.395$, $c_{2}=4.0$, $c_{3}=5.95$, $c_{4}=0.405$.

Supposing that $r_{p}$ is linearly correlated with carbonation degree, then

(20) $$r_{p}=5\times 10^{-8}\left[\left(r_{p,c}-r_{p,0}\right)\alpha_{c}+r_{p,0}\right]$$

where $r_{p,c}$ and $r_{p,0}$ are the peak radius before and after carbonation respectively, and the value of them is determined by *w*/*b*.

Additionally, $p_{v,sat}$, which is temperature dependence in Equation (13) [66] can be expressed by Equation (21).

(21) $$p_{v,sat}=610.8\cdot e^{\frac{17.08085\cdot \tau }{234.175+\tau }}$$

where τ is temperature, °C.

Based on the above, the governing equation of moisture transport that takes the influence of carbonation into consideration is as follows:

(22) $$\frac{\partial \rho_{w}\theta_{w}\left(h\right)}{\partial t}=-\nabla \cdot j_{w,mass}^{car}+I_{w_{e}}$$

where $j_{w,mass}^{car}$ is the flux of moisture considering the influence of carbonation; $I_{w_{e}}$ is the moisture generated by carbonation reaction.

(23) $$j_{w,mass}^{car}=\delta_{c}^{car}\left(h\right)p_{v,sat}\nabla h$$

(24) $$I_{w_{e}}=\kappa I_{ch}M\left(H_{2}O\right)$$

where κ is the correction factor of gas phase water produced by carbonation, being 1.0 in this study; $M\left(H_{2}O\right)$ is the molar mass of water, 18 g/mol.

Then, the following equation is obtained:

(25) $$\rho_{w}\frac{\partial \theta_{w}\left(h\right)}{\partial h}\frac{\partial h}{\partial t}=\nabla \cdot \left(\delta_{c}^{car}\left(h\right)p_{v,sat}\nabla h\right)+\kappa I_{ch}M\left(H_{2}O\right)$$

### 3.3. Chloride Transport

The chloride flux ($j_{c,dif}^{car}$) caused by diffusion after considering the influence of carbonation is expressed as follows:

(26) $$j_{c,dif}^{car}=-D_{c}^{car}\nabla C_{f}$$

where $D_{c}^{car}$ is the diffusion coefficient of free chloride after considering carbonation effect, m^2^/s; $C_{f}$ is the free chloride concentration in pore solution, kg/m^3^ pore solution.

The chloride diffusion coefficient is concerned with the pore structure of concrete, and the $D_{c}^{car}$ with carbonation taken into consideration can be expressed as follows:

(27) $$D_{c}^{car}=D_{c}f_{p}\left(\Omega ,\delta \right)$$

where $D_{c}$ is the chloride diffusion coefficient when carbonation is not taken into consideration, m^2^/s.

(28) $$D_{c}=D_{c,ref}h_{1}\left(h\right)h_{2}\left(T\right)h_{3}\left(t_{e}\right)$$

where $D_{c,ref}$ is the reference chloride diffusion coefficient, which can be obtained through the common formulas, as shown in Equation (29); $h_{1}\left(h\right)$, $h_{2}\left(T\right)$, and $h_{3}\left(t_{e}\right)$ are the influence functions of internal relative humidity, temperature and age on chloride diffusion coefficient, respectively. In addition, according to Ref. [63], the well-acknowledged expressions of the four parameters mentioned above are as follows:

(29) $$D_{c,ref}=\frac{10^{\left[1.776+1.364\left(w/b\right)\right]}+\left[581-1869\left(w/b\right)\right]}{3.1536\times 10^{13}}$$

(30) $$h_{1}\left(h\right)=\left[1+\frac{{\left(1-h\right)}^{4}}{{\left(1-h_{c}\right)}^{4}}\right]$$

(31) $$h_{2}\left(T\right)=exp\left(\frac{U_{C}}{RT_{c,ref}}-\frac{U_{C}}{RT}\right)$$

(32) $$h_{3}\left(t_{e}\right)={\left(\frac{t_{ref}}{t_{e}}\right)}^{m}$$

where $U_{c}$ is the activation energy in chloride diffusion process, and its value ranges from 32.0~44.6 kJ/(mol·K); $T_{c,ref}$ is the reference temperature, 298 K; $t_{ref}$ is the reference age, 28 d; *t_e_* is the actual age, d; *m* is the age reduction factor and is 0.2 in this study.

The chloride flux ($j_{c,w}^{car}$) induced by moisture transport after considering the influence of carbonation can be expressed as:

(33) $$j_{c,w}^{car}=C_{f}V_{w}$$

where $V_{w}$ is the mean moisture velocity in pores, m/s.

Thus, $j_{c}^{car},$ the total chloride flux, can be calculated by the equation below,

(34) $$j_{c}^{car}=j_{c,dif}^{car}+j_{c,w}^{car}=C_{f}V_{w}-D_{c}^{car}\nabla C_{c}$$

In addition, according to the mass conservation equation,

(35) $$\frac{\partial C_{t}}{\partial t}=-\nabla \cdot \theta_{w}j_{c}^{car}=-\nabla \cdot \left(\theta_{w}V_{w}C_{f}-\theta_{w}D_{c}^{car}\nabla C_{f}\right)$$

where $C_{t}$ is the total chloride concentration, kg/m^3^ of concrete.

Furthermore,

(36) $$\theta_{w}V_{w}=j_{w,vol}^{car}=\frac{j_{w,mass}^{car}}{\rho_{w}}=-\frac{\delta_{v}^{car}\left(h\right)}{\rho_{w}}p_{v,sat}\nabla h$$

where $j_{w,vol}^{car}$ is the volume flux of moisture after considering carbonation effect.

Thus, by embedding Equation (36) into Equation (35), a new equation is obtained:

(37) $$\frac{\partial C_{t}}{\partial t}=\nabla \cdot \left(\frac{\delta_{v}^{car}\left(h\right)}{\rho_{w}}p_{v,sat}\nabla hC_{f}\right)+\nabla \cdot \left(\theta_{w}D_{c}^{car}\nabla C_{f}\right)$$

Furthermore, the total chloride content $C_{t}$ is the sum of free chloride in pore solution and bound chloride fixed in matrix,

(38) $$C_{t}=\theta_{w}C_{f}+C_{b}$$

where $C_{b}$ is the bound chloride concentration, kg/m^3^ of concrete.

On the one hand, there is a quantitative relationship between bound chloride and free chloride; on the other hand, bound chloride is closely related to carbonation degree considering the chemical effect of carbonation. Therefore, bound chloride can be considered the function of both free chloride and carbonation degree. Then,

(39) $$\frac{\partial C_{t}}{\partial t}=\frac{\partial \theta_{w}C_{f}}{\partial t}+\frac{\partial C_{b}}{\partial C_{f}}\frac{\partial C_{f}}{\partial t}+\frac{\partial C_{b}}{\partial \alpha_{c}}\frac{\partial \alpha_{c}}{\partial t}$$

Therefore, the updated governing equation of chloride transport is obtained.

(40) $$\frac{\partial \theta_{w}C_{f}}{\partial t}+\frac{\partial C_{b}}{\partial C_{f}}\frac{\partial C_{f}}{\partial t}+\frac{\partial C_{b}}{\partial \alpha_{c}}\frac{\partial \alpha_{c}}{\partial t}=\nabla \cdot \left(\frac{\delta_{v}^{car}\left(h\right)}{\rho_{w}}p_{v,sat}\nabla hC_{f}\right)+\nabla \cdot \left(\theta_{w}D_{c}^{car}\nabla C_{f}\right)$$

Actually, the relation between bound chloride and free chloride can be expressed by three formulas: linear equation, Langmuir equation and Freundlich equation [67]. Considering that the parameter $\alpha_{L}$ (see Equation (41)) can directly reflect the chloride binding capacity of concrete [61], the Langmuir equation was employed in this model:

(41) $$C_{b}=\frac{\alpha_{L}\theta_{w}C_{f}}{1+\beta_{L}\theta_{w}C_{f}}$$

where $\alpha_{L}$ and $\beta_{L}$ are parameters related to concrete mixes. When there is no mineral admixtures, $\alpha_{L}$ = 11.8 and $\beta_{L}$ = 4.0.

Ongoing carbonation will decrease the value of $\alpha_{L}$ since carbonation cripples chloride binding capacity [68,69,70,71]. Supposing that the decrease of chloride binding capacity is linearly correlated with carbonation degree, an equation is obtained:

(42) $$C_{b}=\frac{\alpha_{L}\left(1-d\alpha_{c}\right)\theta_{w}C_{f}}{1+\beta_{L}\theta_{w}C_{f}}$$

where d is the reduction factor of chloride binding capacity due to carbonation.

According to the experimental results presented in Refs. [47,72], chloride binding capacity is almost completely lost in samples of cement-based materials after full carbonation. Therefore, $\alpha_{L}$ is deemed to be zero when concrete is completely carbonated.

(43) $$d=1-\frac{\alpha_{L,c}}{\alpha_{L}}=1$$

where $\alpha_{L,c}$ is the $\alpha_{L}$ of concrete after completely carbonation.

Therefore,

(44) $$\frac{\partial C_{b}}{\partial C_{f}}=\frac{\alpha_{L}\left(1-d\alpha_{c}\right)\theta_{w}}{{\left(1+\beta_{L}\theta_{w}C_{f}\right)}^{2}}$$

(45) $$\frac{\partial C_{b}}{\partial \alpha_{c}}=-\frac{d\alpha_{L}\theta_{w}C_{f}}{1+\beta_{L}\theta_{w}C_{f}}$$

Furthermore, based on Equation (8),

(46) $$\frac{\partial \alpha_{c}}{\partial t}=-\frac{\frac{\partial C_{ch,d}}{\partial t}}{{\left[C_{CaO}\right]}_{0}}=\frac{I_{ch}}{{\left[C_{CaO}\right]}_{0}}$$

### 3.4. Boundary Condition

Since the transports of *CO_2_*, moisture and chloride ions interact with one another, it is necessary to define the boundary conditions of the three simultaneously, and the boundary conditions for wetting process and drying process should be set separately. In the drying process, water and *CO_2_* can exchange at the matrix–atmosphere interface while chloride ions cannot move in or out of the interface. Hence,

(47) $${\mathrm{CO}}_{2}\;\mathrm{transport}:\;C_{CO_{2}}\left(x=0,t\right)=C_{CO_{2}e}$$

(48) $$\mathrm{Moisture}\;\mathrm{transport}:\;h\left(x=0,t\right)=h_{e}$$

(49) $$\mathrm{Chloride}\;\mathrm{transport}:\;(\frac{\delta_{v}^{car}\left(h\right)}{\rho_{w}}p_{v,sat}\frac{\partial h}{\partial x}C_{f}+\theta_{w}D_{c}^{car}\frac{\partial C_{f}}{\partial x}){|}_{x=0}=0$$

where $C_{CO_{2}e}$ is the molar concentration of *CO_2_* in drying environment; $h_{e}$ is the relative humidity of environment. Additionally, Equation (49) shows that chloride flux value is zero at the interface.

In the wetting process, water and chlorides can enter into the interface while *CO_2 cannot_* when specimens contact with salt solution.

(50) $${CO}_{2}\;\mathrm{transport}:\;C_{CO_{2}}\left(x=0,t\right)=0$$

(51) Moisture transport: h(x=0,t)=1.0

(52) $$\mathrm{Chloride}\;\mathrm{transport}:\;C_{f}\left(x=0,t\right)=C_{e}$$

where $C_{e}$ is the chloride concentration of NaCl solution in the external environment.

## 4. Model Solving and Verification

### 4.1. Calculated Results of Chloride Distribution

**Figure 1:**
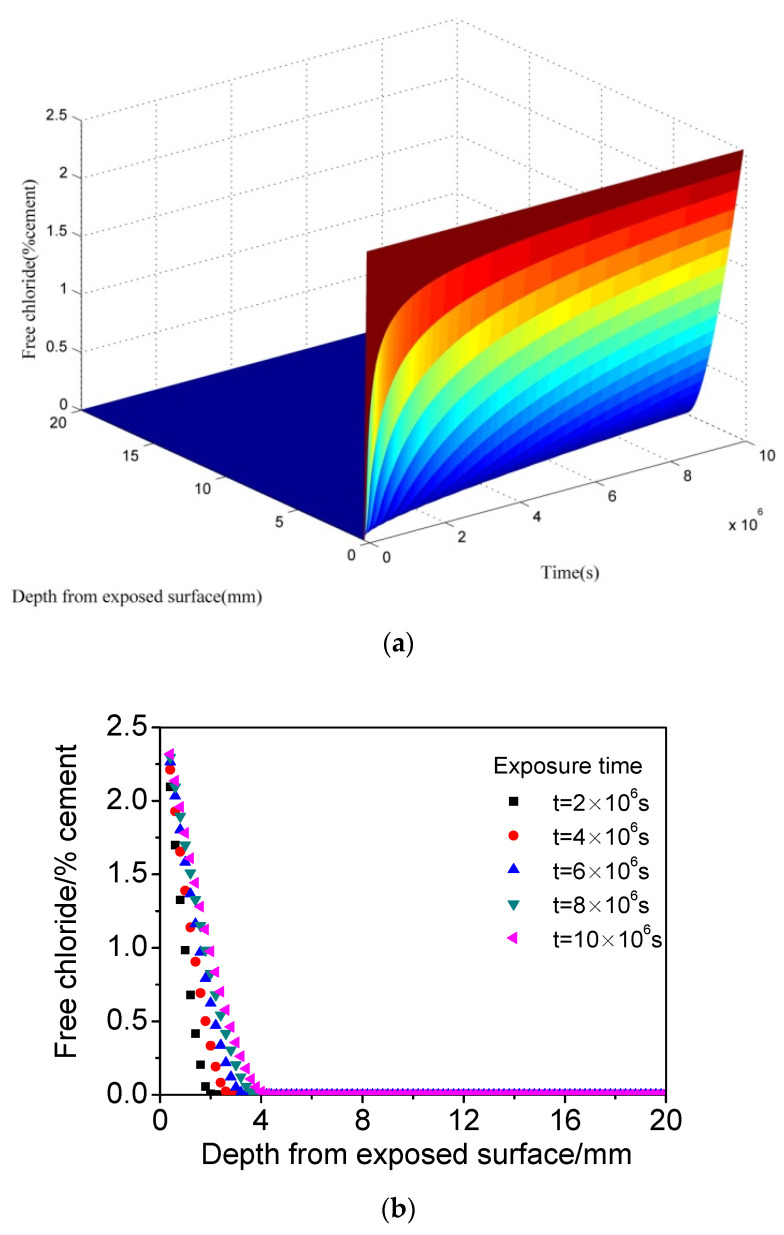
The calculated chloride distribution of concrete induced by diffusion. (**a**) Three dimensions. (**b**) Two dimensions.

The finite difference method was employed to solve the model with the assistance of MATLAB (R2016_win64). Taking concrete of *w/b =* 0.5 as an example, the chloride distribution of concrete exposed to different *CO_2_* concentration was calculated. To highlight the chloride distribution in concrete under cyclic wetting-dying condition, chloride distribution induced by diffusion was also obtained, as shown in Figure 1. It can be observed that chloride decreases monotonously and rapidly with increasing distance from the exposed surface. Additionally, chloride content increases gradually with time.

**Figure 2:**
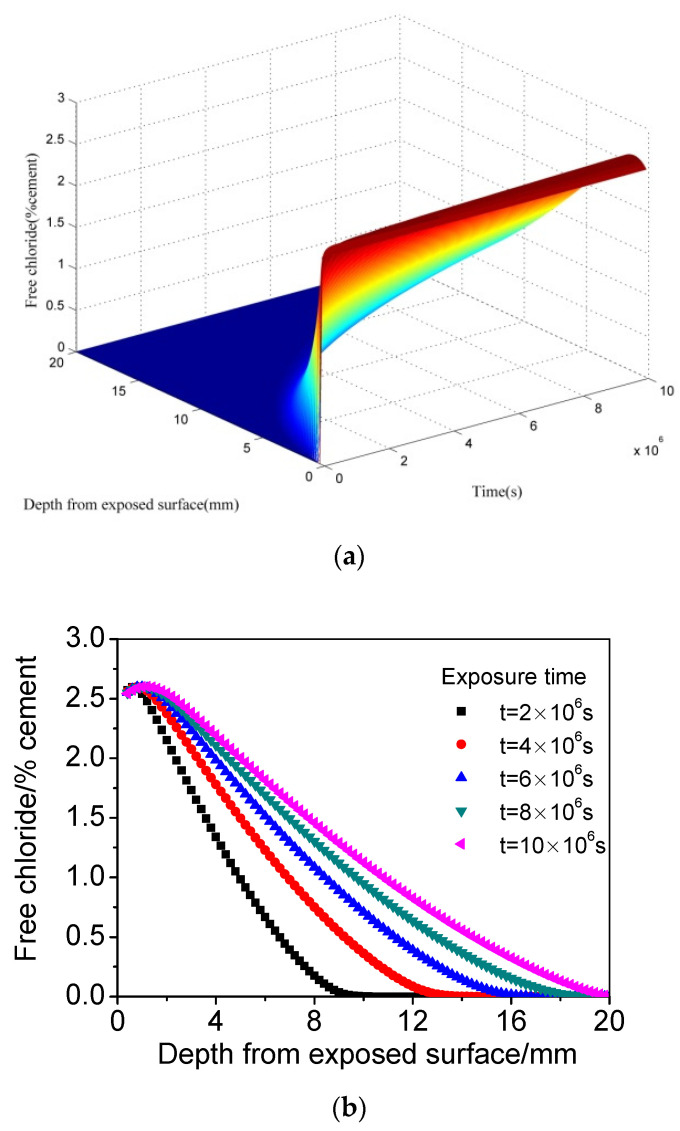
The calculated chloride distribution of concrete subjected to cyclic wetting–drying conditions when the CO_2_ concentration is zero. (**a**) Three dimensions. (**b**) Two dimensions.

Figure 2 presents the calculated chloride distribution of concrete subjected to wetting and drying conditions when the *CO_2_* concentration is zero, meaning that there is no carbonation affecting the transport of chloride in concrete. It can be observed that, compared with the above predicted results (Figure 1), one of the biggest differences is that the chloride maximum phenomenon appears in chloride profiles instead of chloride content decreasing monotonously. The chloride concentration peak forms gradually with increasing exposure time. It can be inferred that capillary suction can also lead to the formation of chloride maximum phenomenon even without the carbonation effect under cyclic wetting–drying conditions, which is consistent with previous studies [8,27,28,29]. Moreover, another prominent difference is that the chloride content after cyclic wetting–drying is significantly higher than that formed only by diffusion with the same exposure time and at the same depth, which, apparently, can be also ascribed to capillary suction.

**Figure 3:**
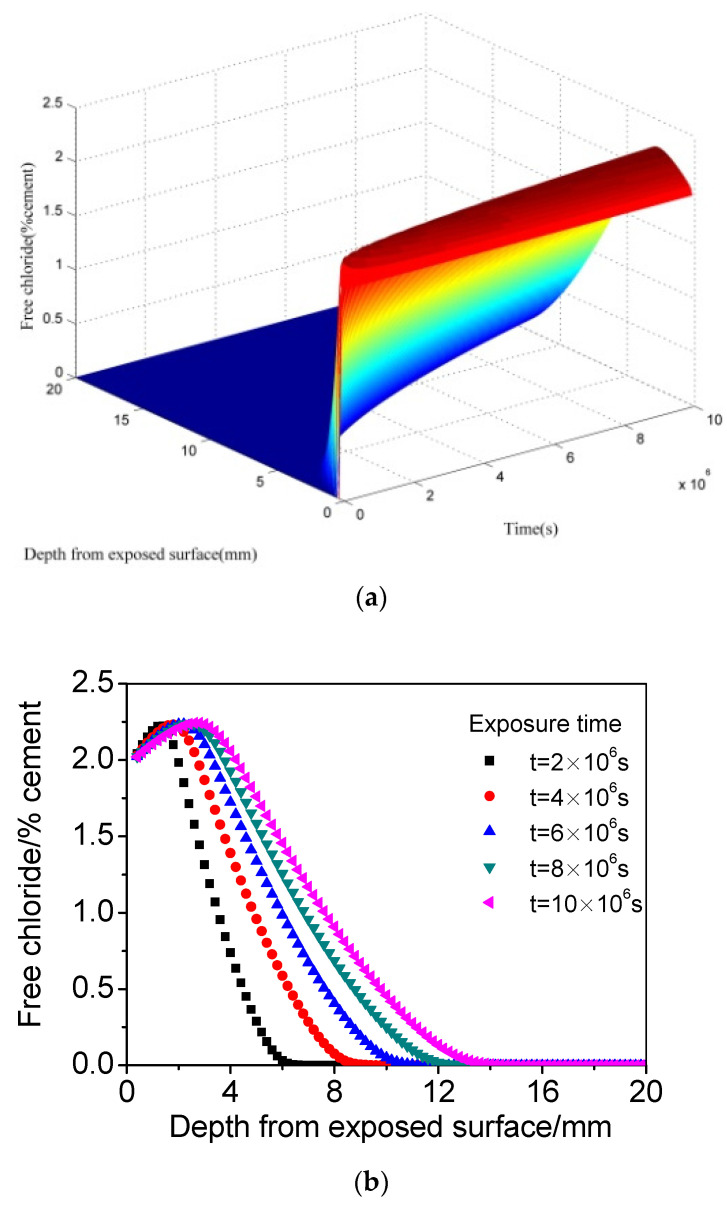
The calculated chloride distribution of concrete subjected to cyclic wetting–drying conditions when the *CO_2_* concentration is 0.04%. (**a**) Three dimensions. (**b**) Two dimensions.

Figure 3 presents the calculated chloride distribution of concrete when the *CO_2_* concentration is 0.04%. The value of 0.04% was adopted to simulate the *CO_2_* concentration in normal-atmosphere wetting–drying environments. It is clear that a significant maximum phenomenon (compared with Figure 2) appears after coupling carbonation. With the reduction of surface chloride content, the chloride concentration peak becomes steep, and the depth at which the peak appears increases at the same exposure time compared with that when carbonation is not considered. Additionally, the chloride concentration peak also becomes more remarkable with increasing time.

**Figure 4:**
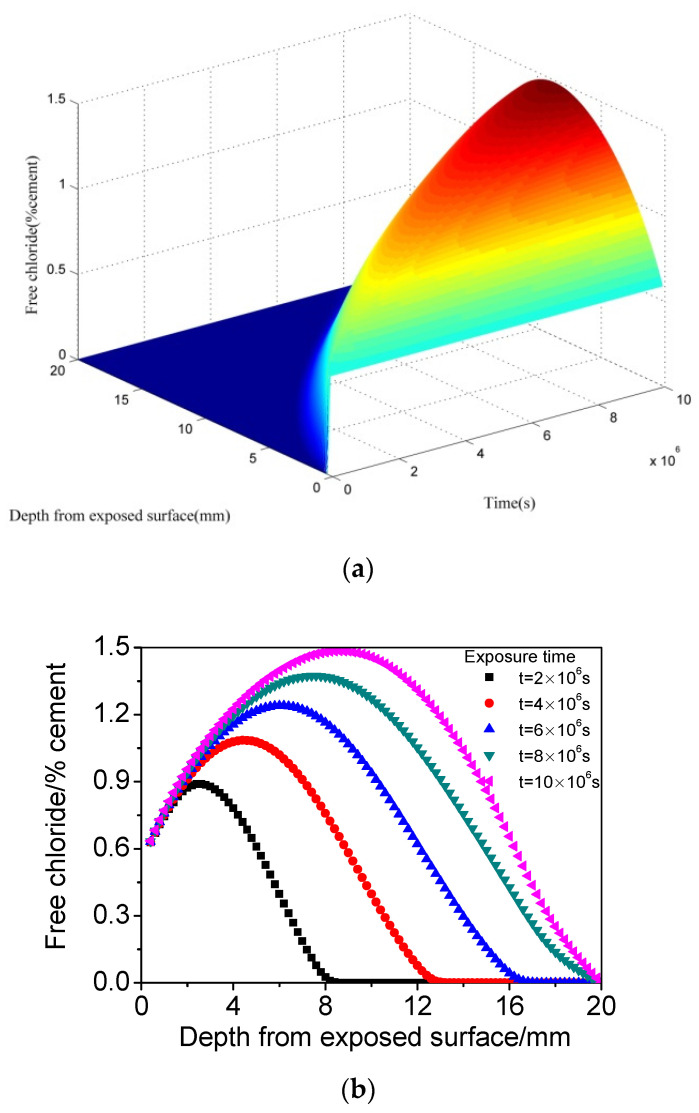
The calculated chloride distribution of concrete subjected to cyclic wetting–drying conditions when the CO2 concentration is 20.0%. (**a**) Three dimensions. (**b**) Two dimensions.

Figure 4 presents the calculated chloride distribution of concrete when the *CO_2_* concentration is 20.0%. The value of 20.0% was adopted to simulate the *CO_2_* concentration in accelerated carbonation wetting–drying conditions. It can be found that with enhanced carbonation effect (compared with Figure 2 and Figure 3), chloride content further decreases, and chloride concentration peak becomes more prominent and broad, and its depth increases greatly, that is to say, the chloride maximum phenomenon becomes more significant. The above calculation results involve both the physical and chemical effect of carbonation on chloride transport, and it can be found that carbonation has a very significant impact on chloride maximum phenomenon. With increasing carbonation degree, although chloride content generally decreases (due to denser pore structure induced by carbonation), the chloride peak protrudes more, and the position where the peak appears becomes deeper (probably related to the chemical effect of carbonation).

### 4.2. Comparison between Experimental Results and Calculated Results

To verify the calculated results, the parameters adopted in the calculation were consistent with those parameters used in the experiment. Some key parameters are presented in Table 3.

**Figure 5:**
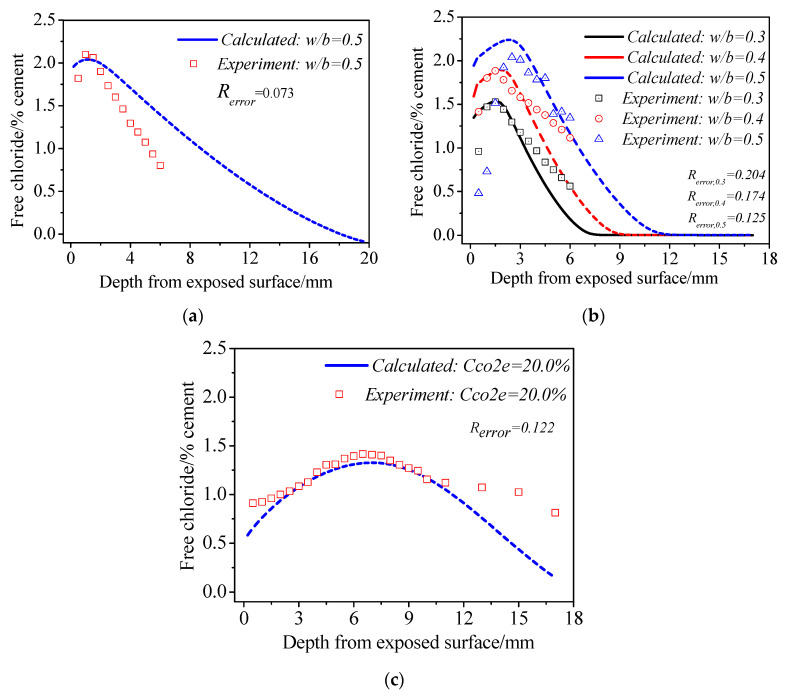
Comparison between experimental results and calculated results: (**a**) *N_2_* condition (*C_CO2e_* = 0); (**b**) normal air condition (*C_CO2e_* = 0.04%); (**c**) accelerated carbonation condition (*C_CO2e_* = 20%).

For the situation of total immersion when only diffusion is considered, numerous experimental studies have verified that chloride content decreases monotonically with increasing depth, and hence the experimental verification of it is omitted here. Then, for the situation of cyclic wetting–drying coupled with different carbonation degrees, Figure 5 presents the calculated and experimental chloride profiles in three situations: no carbonation effect (Figure 5a); normal carbonation effect (Figure 5b); and enhanced carbonation effect (Figure 5c).

As presented in Figure 5a, after specimens were exposed to wetting and drying conditions full of *N_2_*, a chloride concentration peak appeared at 1.0 mm in the chloride profiles, and then the chloride content decreased gradually. The corresponding calculated results also presented a chloride concentration peak at about 0.5~1.5 mm, approximating the experimental results very much. Additionally, the calculated chloride content is generally higher than the experimental content, but their difference is not significant. As shown in Figure 5b, after carbonation is taken into consideration, the chloride maximum phenomenon appears in the chloride profiles of all three *w*/*b* specimens under the normal air condition. The position of the maximum phenomenon is at depth 1.5 mm~3.0 mm, and this increases with *w*/*b*. Meanwhile, the peak chloride concentration also increases with *w*/*b*. The calculated results also successfully predict the appearance of the chloride maximum phenomenon at about the same depth as that obtained in the experiments, and the predicted peak chloride concentration also increases with *w*/*b*. Meanwhile, the chloride content obtained through calculation and the experiment approximate one another very much in places other than the surface area, where the experimental content is lower than calculated content. This suggests that the calculated results basically coincide with the experimental results both in the change law of chloride distribution and in chloride content, which further verifies the reliability of the model built in this research. In addition, it can also be observed that the error coefficients *R_error_* (obtained with Equation (53)) between the simulation results and experimental results under different conditions are all lower than 0.204, with the error of some chloride profiles being less than 0.10, which verifies the accuracy of the model. However, the error coefficient is still not zero, considering that the difference between simulation results and experimental results at different depths varies, which is also one the limitations of the model proposed.

Furthermore, comparing Figure 5a with Figure 5b, it can be observed that the chloride maximum phenomenon under the normal air condition is more significant than that in the *N_2_* condition, both in the calculated results and the experimental results. More specifically, the depth of the maximum phenomenon is greater, and the shape is more prominent. This suggests that, after the coupling carbonation effect, the chloride concentration peak becomes more significant, and its position moves deeper. The authors already claimed in Ref. [11] that the pore structure change induced by carbonation can lead to a decease in overall chloride content, but cannot affect the formation of the maximum phenomenon. Carbonation enhances the chloride concentration peak mainly because of its influence on bound chloride. More precisely, carbonation decomposes and releases surface bound chloride into the pore solution, which increases the total free chloride content in the pore solution, reinforces the drive for chloride to migrate from the surface to deeper positions, and thus facilitates the formation of the maximum phenomenon. In addition, on the other hand, released bound chloride will constantly be taken into deeper positions by capillary absorption, making the position of the chloride peak move further inwards [11]. The finding that the concentration gap between the surface chloride and chloride the peak and the depth of the peak in Figure 5b are higher than those in Figure 5a well supports the analysis above.

Under the accelerated carbonation condition, a more significant chloride maximum phenomenon compared with that in Figure 5b was observed, and the depth of the chloride peak moved further inwards to about 7.0~8.0 mm, which is in good agreement with the calculated results (see Figure 5c). This finding not only confirms the importance of carbonation for chloride distribution, but also verifies the necessity of considering carbonation when simulating chloride transport under cyclic wet–dry environments. In addition to the experimental results of this study, Liu’s [30] findings also verify the simulation results of this model. Liu [30] discovered that chloride content was higher in the normal atmosphere environment, with the chloride peak at a depth of about 3 mm from the exposed surface. After carbonation, chloride content decreased significantly, with the chloride peak (called the convection zone in Liu’s article [30]) at depths of 5~10 mm.

Based on the above, it can be concluded that the significance of the carbonation effect has been identified by the calculated and experimental results. Among numerous simulations of chloride transport under cyclic wetting–drying conditions, Joško’s [27] model successfully predicted the chloride maximum phenomena forming at different depths in chloride profiles, and the calculated chloride content had high accordance with the experimental result. However, the predicted peak location was obviously shallower than that detected in the experiments. The model proposed in this study couples carbonation effect as well as capillary suction, and the calculated results suggest that the depth of the maximum phenomenon moves inwards with increasing carbonation, which coincides with the experimental results. Therefore, the model proposed in this research is further improved and more practical, which is of great significance for promoting the development of chloride transport prediction studies under cyclic wetting and drying environments. However, the data supporting the model coming from the study itself is not enough. Furthermore, no more published data were used to validate the model due to the lack of availability of similar data. Therefore, the accuracy of this model may be hampered, and it needs further exploration.

(53) $$R_{error}=\frac{\sum_{i=1}^{n}\frac{\left|c_{mod,i}-c_{exp,i}\right|}{c_{exp,i}}}{n}$$

where *R_error_* is the error coefficient between modeling data and experimental data of chloride content; *c_exp,i_* is the *i^th^* experimental data of chloride content; *c_mod,i_* is *i^th^* modeling data of chloride content; n is the number of data points. Please note that the exposure time and distance from exposure surface of *c_exp,i_* and *c_mod,i_* are totally the same.

### 4.3. Influence of Key Parameters

#### 4.3.1. External CO_2_ Concentration (*C_co2e_*)

**Figure 6:**
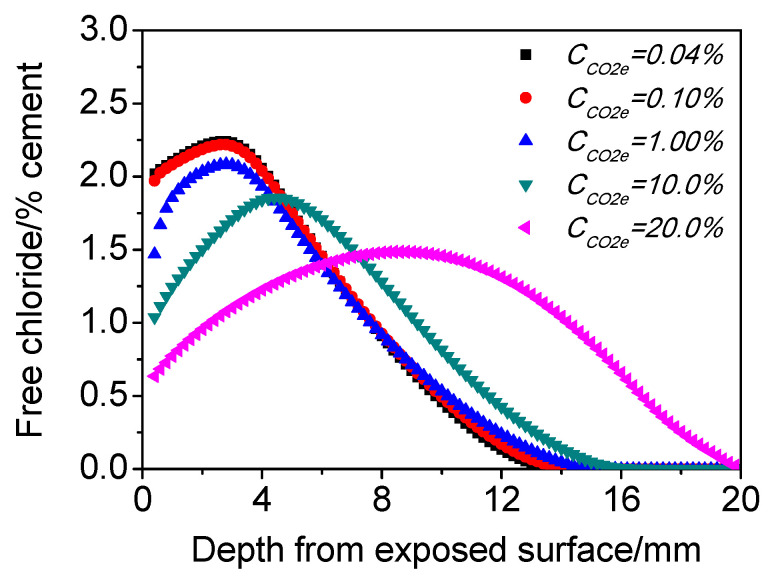
Influence of *C_co2e_* on the chloride distribution of concrete subjected to cyclic wetting and drying conditions.

Figure 6 presents the influence of *C_co2e_* on chloride distribution of concrete exposed to cyclic wetting–drying conditions. It can be observed that different degrees of the chloride maximum phenomena appear in the calculated profiles, corresponding to different *C_co2e_*. With the increasing *C_co2e_*, the depth of chloride peak gets significantly deeper, while chloride content decreases gradually. This means that the maximum phenomenon will become flatter when the *C_co2e_* is higher under cyclic wetting and drying conditions. This result is caused by both the physical and chemical effect of carbonation on chloride transport, which was illustrated in Section 4.2, above.

#### 4.3.2. Environmental Relative Humidity (*h_e_*)

**Figure 7:**
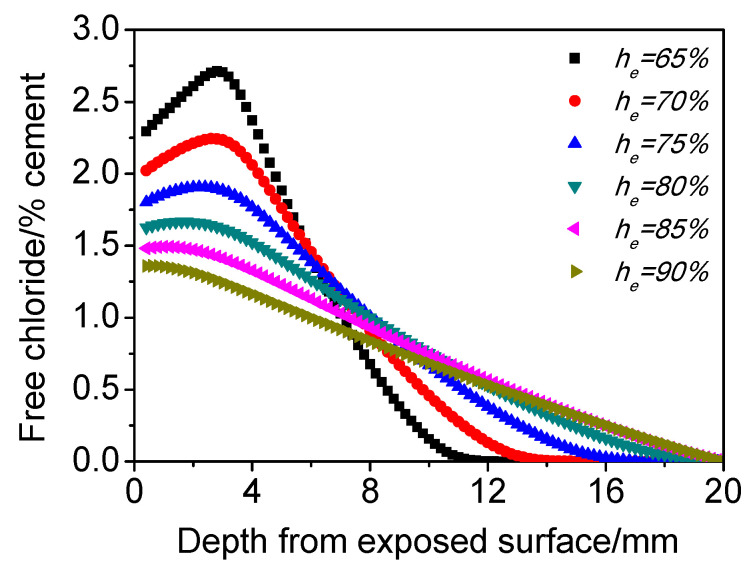
Influence of *h_i_* on the chloride distribution of concrete subjected to cyclic wetting and drying conditions.

Figure 7 shows the influence of *h_e_* on the chloride distribution of concrete subjected to cyclic wetting–drying conditions. It can be found that the lower the *h_e_* is, the more significant the chloride maximum phenomenon is. Both the chloride concentration peak and its depth decrease with increasing *h_e_*, and, especially when *h_e_* reaches 90%, the chloride maximum phenomenon becomes extremely weak. On the one hand, the capillary suction effect becomes weaker with increasing *h_e_*, and on the other hand, although the *C_co2e_* in different-humidity environments is consistent (0.04%) during wetting and drying cycles, the carbonation degree decreases gradually with the increase of humidity [41]. These two factors lead to the weakening of chloride maximum phenomenon with increasing *h_e_*. Moreover, it should be noted that beyond a depth of 8 mm, the smaller the *h_e_* is, the lower the chloride content. This is probably because carbonation happens when *h_e_* is small, which densifies the surface of matrix and blocks the inward diffusion of chlorides.

#### 4.3.3. Environmental Temperature (*T*)

**Figure 8:**
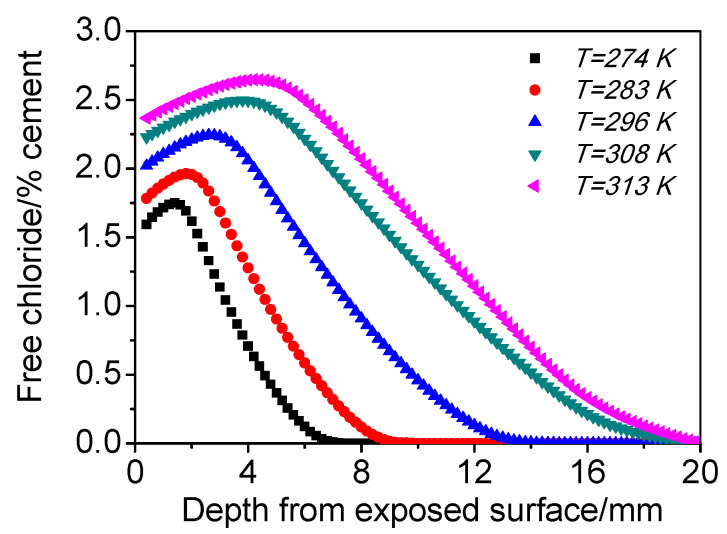
Influence of *T* on the chloride distribution of concrete subjected to cyclic wetting and drying conditions.

Figure 8 shows the influence of T on the chloride distribution of concrete subjected to cyclic wetting and drying conditions. It can be found that the chloride maximum phenomenon becomes very prominent with increasing temperature, that is, the peak chloride content and its depth become greater with increasing temperature. With higher temperature, moisture evaporation is faster, resulting in stronger capillary suction. Meanwhile, *CO_2_* diffusion is more rapid and carbonation range is deeper. As a result, the chloride maximum phenomenon is more significant. In addition, the chloride diffusion rate also increases with temperature, and consequently, the chloride content under higher temperature is higher at all test depths.

#### 4.3.4. External NaCl Solution Concentration (*C_e_*)

**Figure 9:**
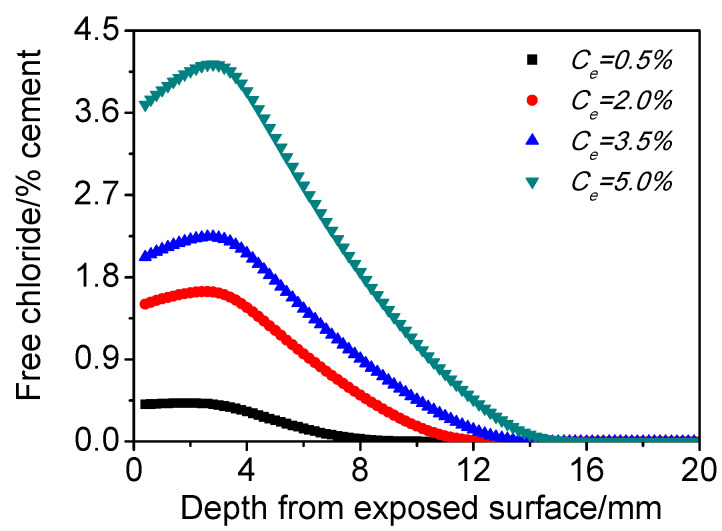
Influence of *C_e_* on the chloride distribution of concrete subjected to cyclic wetting and drying conditions.

Figure 9 presents the influence of *C_e_* on the chloride distribution of concrete under cyclic wetting–drying conditions. It can be seen that chloride content increases with *C_e_* growing within the same time and space, but the depth of chloride concentration peak basically remains the same. This is mainly because the increase of *C_e_* boosts the content of chloride entering into the matrix, although it has very limited influence on carbonation and capillary suction.

#### 4.3.5. Water-to-Binder Ratio (*w*/*b*)

**Figure 10:**
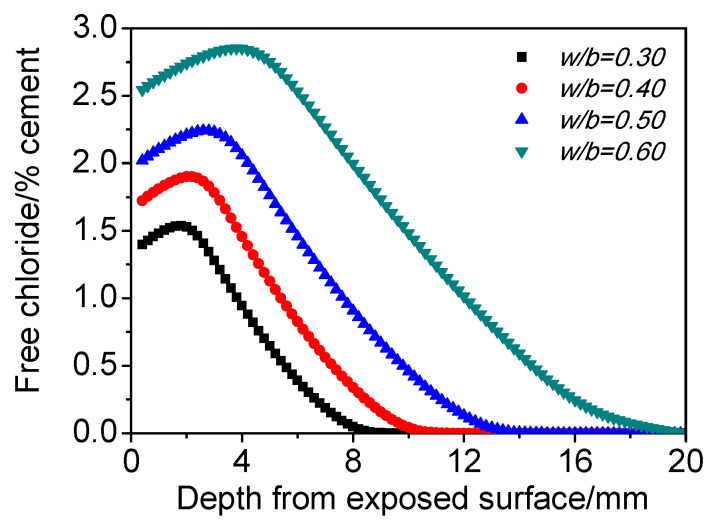
Influence of *w*/*b* on chloride distribution of concrete subjected to cyclic wetting and drying conditions.

Figure 10 presents the influence of *w*/*b* on chloride distribution. It is clear that chloride concentration peak increases with *w*/*b*, and the depth of the peak also increases a bit. In a word, the higher the *w*/*b* is, the more significant the maximum phenomenon is, which was also verified by the experimental results shown in Figure 7. It is generally believed that the higher the *w*/*b* is, the looser the pore structure is. Consequently, it is more convenient for moisture and *CO_2_* to move into and out of matrix, which enhances capillary suction and expands the range of carbonation, leading to a more significant chloride maximum phenomenon.

## 5. Conclusions

1. The results of the chloride transport model coupling carbonation effect show that a chloride maximum phenomenon appears in all predicted chloride profiles of concrete. In addition, the higher the carbonation degree is, the more remarkable the phenomenon is. Specifically, the gap between surface chloride content and the peak chloride content becomes larger and the peak range spans greater. Therefore, carbonation can strengthen the forming of chloride maximum phenomenon under cyclic wetting and drying conditions.
2. The calculated results coincide well with the experimental results under different carbonation conditions, especially in terms of the peak chloride concentration and the corresponding depth.
3. The increase of *w*/*b* and *T* and the decrease of *h_e_* not only boost peak chloride content, they also deepen its depth. Meanwhile, the increase in *C_co2e_*, on the one hand, deepens the depth of chloride peak, and on the other hand, decreases the overall chloride content. Moreover, the growth of *C_e_* significantly increases the peak chloride content but has limited influence on its depth.

## Figures and Tables

**Table 1:** Chemical composition of P.II 52.5 Portland cement.

SiO_2_	Al_2_O_3_	CaO	Fe_2_O_3_	K_2_O	MgO	Na_2_O	TiO_2_
20.0	4.46	63.9	2.99	0.660	0.510	0.110	0.262

**Table 2:** The detailed information of the three exposure conditions.

Conditions	Experimental Procedure for One Testing Cycle	
	Wetting Condition	Drying Condition
N_2_ condition	Wetting in 3.5%NaCl solution for 1 day, 20 ± 1 °C	Drying in a sealed chamber: 20 ± 1 °C, 70 ± 1% RH; 100% N_2_ concentration
Normal air condition	Wetting in 3.5%NaCl solution for 1 day, 20 ± 1 °C	Drying in a room for 6 days: 20 ± 1 °C, 70 ± 1% RH, 0.04% *CO_2_* concentration
Accelerated carbonation condition	Wetting in 3.5%NaCl solution for 1 day, 20 ± 1 °C	Drying in a carbonation chamber: 20 ± 1 °C, 70 ± 1% RH; 20% *CO_2_* concentration

**Table 3:** Values of some key parameters employed in the modeling.

	*C_co_2_e_*/%	*T*/°C	*h_e_*/%	*t*/d	*w*/*b*	*C_e_*/%
***N**_**2**_* **condition**	0	23	70	84	0.5	3.5
**Normal air condition**	0.04	23	70	84	0.3 0.4 0.5	3.5
**Accelerated carbonation condition**	20	23	70	84	0.5	3.5

## Data Availability

The data presented in this study are available on request from the corresponding author.
